# Effect of Different Dietary Inclusion Levels of Sunflower Meal and Multi-Enzyme Supplementation on Performance, Meat Yield, Ileum Histomorphology, and Pancreatic Enzyme Activities in Growing Quails

**DOI:** 10.3390/ani10040680

**Published:** 2020-04-13

**Authors:** Ahmet Engin Tüzün, Osman Olgun, Alp Önder Yıldız, Esra Tuğçe Şentürk

**Affiliations:** 1 Kocarlı Vocational School, Adnan Menderes University, Kocarlı, 09970 Aydın, Turkey; atuzun@adu.edu.tr; 2 Department of Animal Science, Faculty of Agriculture, Selcuk University, Selcuklu, 42130 Konya, Turkey; aoyildiz@selcuk.edu.tr (A.Ö.Y.); esratugcesenturk@gmail.com (E.T.Ş.)

**Keywords:** sunflower meal, enzyme, quail, performance, histomorphology, enzyme activity

## Abstract

**Simple Summary:**

In the poultry industry, where the cost of feed constitutes most of the expenses, sudden changes in feed prices make it a challenge for nutritionists to maintain the yield and health of animals while managing the diet costs. It is the first solution that comes to mind to use inexpensive raw materials while preparing diets in practical conditions. In addition, feeds used in diets should be digestible by poultry. In the poultry digestive system, feeds such as sunflower meals are difficult to digest due to containing high cellulose because there is no cellulase secretion in the digestive system. The use of multi-enzyme which enhanced the digestibility of nutrients and decreased antinutritional compounds such as cellulose and nonstarch polysaccharides in diets with sunflower meal can positively affect performance and the profitability of the growers.

**Abstract:**

This study aimed to investigate the effect of dietary sunflower meal (SFM) and multi-enzyme levels on performance, carcass traits, intestinal histomorphology and pancreatic enzyme production in quails. Three hundred and twenty, 1-day-old quail chicks were divided into 8 groups with 4 replicates consisting of 10 birds each in the group. The experiment was randomized design consisting of a 4 × 2 factorial arrangement, with four levels of SFM (0%, 10%, 15%, or 20%) and two levels of multi-enzyme (0.0 or 1.0 g/kg) inclusion in the diet. The body weight, body weight gain, and feed conversion ratio were negatively influenced by the 15% and 20% SFM (*p* < 0.01) but were not affected by the 10% SFM for 6 week age. The relative gizzard (*p* < 0.05) weights significantly increased with 20% SFM, but the relative breast weight decreased (*p* < 0.01). The relative liver weight increased by the addition of enzymes in the diet (*p* < 0.05). The villus width (*p* < 0.01) and villus surface area (*p* < 0.05) of ileum increased linearly with SFM, whereas the villus height (*p* < 0.01), villus height: crypt depth (*p* < 0.01) and tunica muscularis thickness (*p* < 0.01) decreased linearly with SFM. Consequently, it is possible to say that the birds with the least absorptive same weight are the most efficient. The addition of multi-enzyme increased villus height and crypt depth but decreased tunica muscularis thickness of ileum (*p* < 0.01). Chymotrypsin activity in the pancreas decreased linearly with SFM (*p* < 0.01). Amylase activity in the pancreas decreased significantly with the addition of the multi-enzyme (*p* < 0.05). As a result of the study, SFM can be used at a 10% level in growing quail diets with beneficial effects on the absorption surface area. The effects of enzyme supplementation on parameters measured were less pronounced than the SFM inclusion level that higher villus height and lower tunica muscularis thickness were determined in multi-enzyme-fed birds compared to those untreated counterparts.

## 1. Introduction

Soybean meal is traditionally used over decades as the main source of protein in the nutrition of poultry with its high protein and lysine content and little amount of anti-nutritional compounds as well. Moreover, most of the soybean production in the world consists of genetically modified soybeans and its use in human and/or animal nutrition is planned to be banned in many countries. Therefore, the need for alternative protein sources such as lupine, chickpea, faba bean, and insect meal is increasing day by day [1,2,3,4]. The high price of soybean meal increases the importance of sunflower meal (SFM), which is one of the most produced oilseed meal in our country, as an alternative protein source to soybean meal [5]. In other words, the SFM can be an economical source of protein in countries where soybean is uncultivated or little cultivated. The use of SFM, the oil industry by-product, in broiler diets is limited due to its high cellulose and low lysine content but it is free of anti-nutritional compounds and contains high calcium, phosphorus, and methionine [6].

While some researches indicated that SFM can be used up to 15% in broiler diets without negatively affecting performance [7,8], some studies reported that it can be used at higher levels [9,10], especially with the addition of enzymes [11]. On the contrary, there were studies reported that the use of 10% and more SFM in broiler diets negatively affected the growth performance [12,13,14,15]. Additionally, Karayagız, and Bulbul [16] reported that SFM can be used up to 20% in growing quail diets without negatively affecting performance.

The SFM contains 4.5% soluble and 23% insoluble nonstarch polysaccharide (NSP) [17]. However, the digestive system of monogastric animals has poor potential to produce enzymes that can digest NSP such as cellulose, glucan, xylan, so the use of exogenous enzymes in the diets of these animals is important in terms of NSP digestion [18,19]. Therefore, the addition of enzymes to diets containing raw materials such as SFM containing NSP increases digestion and consequently improves the performance of the birds. Bilal et al. [11] stated that the addition of enzymes to the diet containing 20% SFM improved the performance of broilers.

Studies investigating the effects of graded levels of SFM with or without multi-enzyme on growing quail performance, histomorphology of intestine and digestive enzyme activities, to the best our knowledge are few. Furthermore, there is no study concerning pancreatic enzyme response to SFM in growing quail diets. Therefore, this study was carried out to determine the effects of different levels of SFM and multi-enzyme in the diets on performance, carcass traits, intestinal morphology, and pancreatic enzyme activities in growing quails.

## 2. Materials and Methods

### 2.1. Ethical Approval

The animal care practices used in the experiment were in accordance with animal welfare rules stated in Article 9 in government law in Turkey (No. 5996).

### 2.2. Animals and Experimental Feeds

A total of 320 one-day-old Japanese quail chicks were assigned to eight dietary treatments with four replicates each with ten chicks, in a 4 × 2 factorial arrangement by randomized design. Treatments consisted of four levels (0%, 10%, 15%, or 20%) of SFM and two levels (0.0 or 1.0 g/kg) of multi-enzyme. Treatment diets were formulated according to NRC [20] (Table 1). The SFM used in the experiment contained 909 g dry matter/kg feed, 375 g crude protein/kg feed, 167 g crude fiber/kg feed, and 73 g crude ash/kg feed. The exogenous enzyme mixture contains xylanase, protease, α-amylase, cellulase, and hemicellulase enzymes. The temperature of the experimental house without windows was set at 30 and 26 °C for the first and second-sixth week of age, respectively, with a relative humidity of 55% ± 5. The lighting program was 23 h L: 1 h D with a light intensity of 8 lux/m^2^ throughout the experimental period. The quail chicks were housed (45 × 30 × 20 cm) in electrically heated battery cages and were offered water and feed ad libitum from 0 to 6 week.

### 2.3. Determination of Performance Parameters

Throughout the experiment, body weight (BW) and feed intake (FI) of quails on each replicate are measured by the precision with 1 g delicate (Jadever^®^, Fujian, China) as g/birds on the 0th, 3rd and 6th week of age. The body weight gain (BWG) of birds were calculated from these measurements. The feed conversion ratio (FCR) was calculated with the formula FI (g)/BWG (g).

### 2.4. Determination of Relative Weight of Carcass and Some Organs

At 6 week of age, two male quails from each subgroup were euthanized by cervical dislocation and then the carcass and cut-up parts, liver, heart, gizzard, and proventriculus were weighted by a precision scale with 0.01 g delicate (Kern&Sohn GmbH, Balinge, Germany) for the determination of carcass yield and relative organ weights. The carcass and organ relative weights were calculated as% of body weight and thigh + drumstick and breast relative weights were calculated as% of carcass weight. Samples were also collected from the pancreas and ileum for the further enzyme and histological analysis, respectively.

### 2.5. Determination of Histomorphometry of Ileum

For ileal histomorphological measurements from the ileum was exenterated and immediately were removed fixed in 10% buffered formalin for 72 h. The intact and well-oriented crypt-villus units of each sample were divided into triplicate cross-section. The preparation and fixation procedures and subsequent measurements for the villus and crypts dimension were carried out to protocol designated by Xu et al. [21]. The villus height (VH) was measured from the crypt-villus junction to the brush border at the tip. Villus width (WW) was measured between brush borders of opposing epithelial cells at the midpoint of the villus where possible. Crypt depths (CD) were taken at the level of the basement membranes of opposing crypt epithelial cells. The ratio between VH and CD was described as VH/CD. The tunica muscularis thickness (TMT) was defined as the distance between the lamina muscularis mucosae internally and the tunica serosa externally. Villus surface area (VSA) was calculated using formula = (2π) × (villus width/2) × (villus height) as described by Sakamoto et al. [22].

### 2.6. Determination of Digestive Enzyme Activities

The pancreas was harvested from each quail within 5 min after death, placed in aluminum foil, snap-frozen in liquid nitrogen, and stored at −80 °C. The following procedures were according to the method described by Chen et al. [23]. Amylase activity (EC 3.2.1.1) was determined according to Bernfeld [24], where the reducing groups liberated from starch are measured by the reduction of 3,5-dinitrosalicylic acid (Sigma Chemical Co., St. Louis, MO, USA). One-unit activity was defined as the amount of enzyme, which liberates 1 micromole of glucose from soluble starch per minute at 37 °C and pH 6.9 under the specified conditions. Activation of chymotrypsin to chymotrypsin (EC 3.4.21.1) was based on the method of Glazer and Steer [25]. Chymotrypsin activity was determined according to Hummel [26] by measuring an increase in absorbency at 256 nm resulting from the hydrolysis of benzoyl-L-tyrosine ethyl ester (BTEE, Sigma Chemical Co.). One unit of chymotrypsin activity was defined as 1 mmol of BTEE hydrolyzed per min in pH 7.8 phosphate buffer at 37 °C. The lipase activity was determined according to the method of Sigurgisladottir et al. [27] with slight modification. Briefly, 0.1 mL of enzyme preparation is mixed with 0.1 mL of *p*-nitrophenyl laurate (pNPL) (10 mM in ethanol) solutions. The mixture was incubated for 30 min at 65 °C and then mixed with 0.25 mL of Na_2_CO_3_ solution (0.1 M). After centrifugation at 10,000× *g* for 15 min, the absorbance of the supernatant was measured by using a spectrophotometer at 410 nm. One lipase activity was determined as the amount of enzyme that caused the release of 1.0 μmol of *p*-nitrophenol in 1 min under the experimental conditions. All enzyme activities were measured spectrophotometrically (Multiskan GO spectrophotometer, Thermo Scientific, Waltham, MA, USA).

### 2.7. Statistical Analysis

The data were subjected to ANOVA using the General Linear Model (GLM) procedure in Minitab [28] and differences between the group means were determined by Duncan’s multiple range tests. Additionally, linear and quadratic effects of sunflower meal levels were determined by using orthogonal contrasts. Statements of statistical significance were based on a probability of *p* < 0.01 or *p* < 0.05.

## 3. Results

### 3.1. Performance Parameters

The BW, BWG, FI and FCR results are shown in Table 2. No significant effect of multi-enzyme as the main factor and SFM × enzyme interaction were noted on BW, BWG, FI, FCR or mortality (data not shown) in growing quail (*p* > 0.05). The SFM did not significantly affect FI from 3 to 6 or 0 to 6 week periods (*p* > 0.05). Throughout the experiment, the BW and BWG decreased usually with increasing levels of SFM, especially at 15% and 20% supplementation levels. Similarly, FCR of growing quail was worsened linearly by increasing levels of SFM in the diet. Between 0 and 3 week of age, FI was increased with supplementation of SFM at the levels of 15% and 20% (*p* < 0.05). However, FI in growing quail were barely affected by SFM supplementation at 3 to 6 or 0 to 6 week periods (*p* > 0.05). Throughout the experiment (0 to 6 week), the highest BW, BWG and the best FCR were obtained in the no SFM added control group, while the lowest BW, BWG, and the worst FCR were observed in the birds fed 20% SFM.

### 3.2. The Relative Carcass and Organ Weights

The relative weight of carcass, cut-up parts and some organs are presented in Table 3. Graded levels of SFM inclusion in quail diet with or without multi-enzyme supplementation did not affect relative carcass and pancreas weights (*p* > 0.05). The relative breast weight was linearly decreased by increasing levels of SFM in the diet (*p* < 0.001). In addition, relative thigh + drumstick (*p* < 0.01), liver (*p* < 0.01), heart (*p* < 0.01) and gizzard (*p* < 0.05) weights were influenced linearly by SFM. The highest relative thigh + drumstick, gizzard and heart weights were obtained with 20% SFM supplementation. The highest relative liver weight was determined in quails fed diets containing 10% SFM. The addition of multi-enzymes to growing quail diets significantly increased relative liver weight (*p* < 0.05) but not the other slaughter measured (*p* > 0.05). The interaction between SFM and multi-enzyme supplementation significantly affected relative breast (*p* < 0.05), liver (*p* < 0.01), heart (*p* < 0.05), gizzard (*p* < 0.05), and proventriculus (*p* < 0.05) weights of growing quails. The relative breast weight was significantly higher in the group fed on a diet containing 0% SFM × 0.0 g/kg enzyme than in groups fed on with 15% × 0.0 g/kg and 20% × 0.0 g/kg. The highest relative liver weight was obtained in the 10% × 0.0 g/kg and 10% × 1.0 g/kg treatment groups, and the differences between these groups and the 0% × 0.0 g/kg, 15% × 1.0 g/kg and 20% × 0.0 g/kg groups were statistically significant. The relative heart and gizzard weights in 20% × 1.0 g/kg group were significantly higher than 15% × 0.0 g/kg and 15% × 1.0 g/kg and 0% × 0.0 g/kg, 10% × 1.0 g/kg and 15% × 1.0 g/kg groups, respectively. The lowest relative proventriculus weight was obtained in the 0% × 0.0 g/kg treatment group, and the differences between this group and the 0% × 1.0 g/kg were statistically significant.

### 3.3. Histomorphology Parameters of Ileum

The villus width (VW), villus height (VH), crypt depth (CD), villus height/crypt depth (VH/CD), tunica muscularis thickness (TMT) and villus surface area (VSA) are presented in Table 4. Dietary SFM significantly affected all of the histomorphology parameters of ileum in growing Japanese quails. There was a significant effect of SFM on VH (*p* < 0.001) that the VH was decreased by 10% and 20% of SFM supplementation. The CD was decreased by 10% SFM level, but it was increased by 15% SFM level. The VH/CD linearly decreased with the increased SFM supplementation (*p* < 0.001). The VW increased significantly with SFM supplementation and maximized by 10% SFM level (*p* < 0.001). The VSA was increased by 10% SFM addition to the diets (*p* < 0.05) as compared to those containing 0% and 20% SFM (*p* < 0.05). The TMT decreased with 10% and 15% SFM supplementation to the diets (*p* < 0.01) as compared to those 0% and 20% SFM levels. The addition of multi-enzyme to the diet had significant effects on VH, CD and TMT (*p* < 0.001), but did not influence other parameters (*p* > 0.05). Multi-enzyme supplementation to diet markedly increased (*p* < 0.01) the VH and CD of ileum, whereas the TMT of ileum significantly (*p* < 0.01) decreased by the addition of multi-enzyme in growing quails. The SFM × enzyme interaction significantly affected histomorphological traits in the ileum (*p* < 0.05). The VW and VSA were significantly higher in the 10% × 1.0 g/kg group and the CD was significantly higher in the 15% × 1.0 g/kg group compared to other experimental groups. The lowest VH was obtained in the 10% × 0.0 g/kg group compared with the other groups, except for the 20% × 1.0 g/kg group. The TMT of ileum was also found to be lower in the quails fed the diet containing 10% × 1.0 g/kg and 15% × 1.0 g/kg than other groups, except for 20% × 1.0 g/kg group.

### 3.4. Enzyme Activities of Pancreas

The amylase, lipase, and chymotrypsin activities of the pancreas are presented in Table 5. The dietary inclusion level of SFM did not affect the amylase and lipase activities in the pancreas. In this experiment, the chymotrypsin activity of the pancreas decreased linearly (*p* < 0.001) by the dietary SFM levels, reaching a minimum with a level of 20% SFM in the diet. The addition of 1.0 g/kg multi-enzyme to the diet significantly reduced (*p* < 0.05) amylase activity in growing quails but did not affect the lipase and chymotrypsin activities in the pancreas. The interactions between SFM and multi-enzyme supplementation significantly affected amylase (*p* < 0.05) and lipase activities of the pancreas but did not affect chymotrypsin activity (*p* > 0.05) in growing quails. The amylase activity was significantly higher in the 0% × 0.0 g/kg group compared to the 0% × 1.0 g/kg and 10% × 1.0 g/kg groups. The lipase activity was higher in the 0% × 0.0 g/kg group compared to the 0% × 1.0 g/kg group.

## 4. Discussion

### 4.1. Performance Parameters

The results of previous studies stated that SFM could be included up to 15% [8,19] and 20% levels [10,11] in the diet with no negative effects on performance parameters such as BWG or FCR in broilers. In addition, Christaki et al. [29] noted that the BW, FI, and FCR of quails were not significantly affected by dietary SFM ranging from 3.5% to 6.5% levels. In the present study, on the contrary, there was a linear decrease in BW, BWG, and FCR with increased SFM supplementation, especially at 15% and 20% levels. The deterioration in these parameters is more evident at the 20% SFM level. Similarly, Araujo et al. [14] showed that increasing levels of SFM (from 8% to 24%) in the diet decreased BWG and worsened FCR of broilers. In addition, Bilal et al. [11] demonstrated that FCR in broilers was negatively affected by high cellulose in the diet. The deterioration of performance with SFM at the level of 15% and 20% in growing quails in the present study may be caused by high crude cellulose contents in these diets. The SFM contains highly insoluble cellulose. Due to the rapid passage of insoluble cellulose through the digestive system, and the digestive system of poultry is short, so digesta stays in the intestine for a short time and consequently leads to low absorption of nutrients required for growth [30]. However, broilers fed with a high level of SFM can compensate for low nutrient absorption by increasing FI [14]. In the current study, FI of quails increased with SFM in the diet for 0–6 week periods, but it was statistically insignificant (*p* = 0.052). Therefore, it can be said that the digestive system of quails does not have FI capacity to consume enough feed to prevent a decrease in growth when fed diets with high fiber. In this study, the addition of multi-enzyme to the SFM diets had no significant effect on performance parameters in growing quails. These results correlate well with Araujo et al. [14] but disagree with Bilal et al. [11] who reported that the addition of NSPase to diets containing up to 20% SFM level improved FCR in broilers. Similarly, Horvatovic et al. [15] noted that the supplementation of enzymes to the diets containing 6% or 8% levels of SFM in the grower phase significantly improved performance in broilers. Similar results have been reported by Kırkpınar and Basmacıoğlu [5], Tavernari et al. [10], and Mushtaq et al. [31] with regard to the addition of enzymes to the broiler diets including 15% to 35% SFM. The difference between the results of the current experiment and previous studies in terms of the effect of adding enzymes to SFM-containing diets on performance may be due to the properties of SFM and enzymes, species of bird and the ingredient composition of experimental diets. The distribution rates of female quails in the interaction groups are 40%, 47.5%, 47.5%, 50%, 45%, 52.5%, 55%, and 45%, respectively. In this study, we think that male/female quail proportions in the experiment groups are not a distribution that will affect the results.

### 4.2. Relative Carcass and Some Organ Weights

In the present study, relative thigh + drumstick, breast, liver, heart and gizzard weights were linearly influenced by dietary SFM levels and the highest relative thigh + drumstick, heart and gizzard weights, and the lowest relative breast weight were noted at 20% SFM in the diet. The highest relative liver weight was obtained at the level of 10% SFM in the diet. These findings disagree with Christaki et al. [29] who reported that dietary SFM levels did not affect carcass parameters and relative organ weights in quails. Similar results have also been reported by Araujo et al. [14] and Horvatovic et al. [15] in broilers. However, Moghaddam et al. [7] stated that dietary SFM levels in broilers affected the relative thigh, liver and gizzard weights, but did not affect the relative breast weight. Alagawany et al. [19] noted that the carcass traits of broilers was not affected by dietary SFM levels except for the relative heart weight, which was decreased with dietary SFM supplementation. In addition, Bilal et al. [11] demonstrated that dietary SFM level did not affect carcass parameters and relative organ weights, but did improved gizzard weight in broilers. No significant effect was found for the multi-enzyme supplementation on carcass parameters except for relative liver weight which was increased by supplementation of multi-enzyme to the quail diets. These results partially agree with Mushtaq et al. [31] and Alagawany et al. [19] who demonstrated that all carcass parameters were not affected by the addition of enzymes in broiler diets. The effect of interactions between dietary SFM level and multi-enzyme addition on relative breast, liver, heart, gizzard, and proventriculus weights in quails were statistically significant, but this was not the case for other parameters measured (*p* > 0.05). These findings are partially in line with Alagawany et al. [19] who showed no effects of dietary SFM level, with or without enzyme addition, on carcass parameters except for carcass and dressing rates in broilers. In addition, Bilal et al. [11] reported that the relative thigh, liver, and gizzard weights were influenced by interactions between dietary SFM level and enzyme addition in broilers. Amerah et al. [32] reported that all studied carcass parameters were not affected by interactions between dietary SFM level and enzyme addition in broilers. Similar results have also been reported by Horvatovic et al. [15] in broilers. While there is a match between the results of the current study and the results of some previous studies, there is a discrepancy with others.

### 4.3. Histomorphology Parameters of Ileum

Dietary SFM levels linearly or quadratically affected all histomorphological parameters of the ileum. The VW was significantly lower in the control group in comparison with the other groups. The VH was significantly higher in the control group compared with diets containing 10% and 20% SFM levels. The CD was linearly affected by the SFM level and minimized at the 10% SFM level and maximized at the 15% SFM level. The VH/CD and TMT showed a linear and quadratic decrease, respectively, with increasing SFM levels in the diet, and both parameters were minimized at the level of 15% SFM. The VSA of the 10% SFM group was higher compared to the control and 20% SFM groups. The result partially disagrees with the findings of Moghaddam et al. [7], who stated that dietary SFM level (7%, 14%, and 21%) did not affect the VW, VH, CD, and VH:CD of the ileum, but these parameters in the duodenum and jejunum were affected linearly or quadratically by dietary SFM levels in broilers. In addition, Oliveira et al. [33] noted that the villus height and crypt depth of the ileum were significantly reduced by the increased SFM level (from 4% to 16%) in the diet. The VH and CD were higher and TMT was lower in quails fed the multi-enzyme supplemented diet than those fed the non-supplemented diet. Similarly, Oliveira et al. [33] reported that the addition of enzymes to the diets supplemented with SFM increased the VH and CD in broilers. In this study, the histomorphological parameters of ileum statistically interacted between dietary SFM level and multi-enzyme in growing quails. In the literature, only one study was found reporting an interaction between dietary SFM and enzyme in terms of intestinal histomorphology, and it was reported that dietary SFM with or without enzyme had a statistically significant effect on the VH and CD of the duodenum, jejunum, or ileum in broilers [33]. Crypts are the regions where villus is produced, and the increase of CD indicates that epithelial cell (villus) production is high. The villus are where the absorption of nutrients takes place in the small intestine. Contrary to the increase of VW, the increase of the height of the villus indicates that the absorption surface increases, so that absorption of nutrients is more effective [34]. In the current study, there was an irregular decrease in VH and TMT with dietary SFM levels. As a hypothesis, it can be said that the high cellulose derived from SFM causes the rapid passage of intestinal content and the mucosal structure of intestinal tissue is damaged, and thus the absorption of nutrients is impaired due to the decrease in the height of the villus. In addition, the thinning of the TMT may be due to the thinning of the mucosa structure, which is an indicator of intestine health, but this should be examined by further studies. Furthermore, the energy and protein required for intestinal maintenance are higher than other organs and tissues, and this amount can reach 12% of total nutrient consumption in fast-growing broilers [7,35]. The negative effect of dietary SFM on performance may be due to the diminishing of nutrients absorption and needing more nutrients for the regeneration of intestinal tissue by its negative effects on gut health. Although the addition of multi-enzyme to diets containing SFM improved VH and CD, this improvement was not reflected in performance.

### 4.4. Enzyme Activities of Pancreas

The chymotrypsin production in the pancreas was linearly affected by dietary SFM levels and showed a minimum with 20% SFM supplementation. There are no studies in the literature about the effect of dietary SFM levels on enzyme activities of the pancreas. However, Salari et al. [36] and Moghaddam et al. [7] reported that dietary full-fat sunflower seed (7%, 14%, or 21%) or SFM (7%, 14%, or 21%) levels did not affect ileal protease activity in broilers. On the other hand, Alagawany et al. [19] stated that the SFM substitution of 75% of soybean meal in the diet significantly reduced ileal protease activity in broilers. In earlier studies, it was noted that dietary cellulose levels caused a decrease in pancreatic chymotrypsin activity in rats [37]. In the later studies carried out in broilers, there were contrary statements. For example, Bogulsawska-Tryk [38] showed that increasing the level of cellulose in broiler diets increases pancreatic chymotrypsin activity. Interestingly, however, Botermans and Pierzynowski [39] showed that there was a positive correlation between body weight and pancreatic secretion in growing pigs. In the current study, a significant decrease in the body weight of quails was observed with a high SFM level in the diet. In parallel, there was a decrease in the chymotrypsin activity of the pancreas at the same SFM levels. We hypothesized that there could be a positive correlation between body weight and proteolytic enzyme activities in the pancreas, and that high SFM levels (15% and 20%) in the diet cause low body weight as a consequence of reduced chymotrypsin activity in the pancreas.

The addition of multi-enzyme to quail diets with different levels of SFM only affected the amylase activity of the pancreatic enzymes examined, and the addition of multi-enzyme significantly decreased the amylase activity of the pancreas. This result agrees with Yaghobfar and Kalantar [40] who stated that the addition of exogenous enzymes blend to the diet containing barley- or wheat-based decreased pancreatic amylase activity in broilers. However, Alagawany et al. [19] reported that the addition of enzyme to broiler diets containing full-fat sunflower seeds increased ileal amylase activity. In addition, Engberg et al. [41] reported that the addition of xylanase to wheat-based broiler diets does not affect the amylase activity of the pancreas but increases lipase and chymotrypsin activities of the pancreas. The reason for the decrease in amylase production in the pancreas with the addition of enzymes to the diet may be due to it has increased the amount of amylase and/or has reduced the amount of amylose in the gut by the exogenous enzymes, especially amylase. Thus, this may have stimulated the feedback of the pancreas for amylase production and caused a decrease in pancreatic amylase activity.

Interactions between dietary SFM level and enzyme addition had an important effect on amylase and lipase activities in the pancreas. The highest and lowest values in both enzymes were determined in the 0% × 0.0 g/kg and 0% × 1.0 g/kg groups, respectively. Similarly, Alagawany et al. [19] reported that interactions between SFM level and enzyme addition have an important effect on ileal enzyme activities in broilers.

## 5. Conclusions

In conclusion, the use of SFM at levels of 15% and more in growing quail diets negatively affected performance and pancreatic chymotrypsin activity. In addition, high SFM levels partially negatively affected ileum histomorphology. The addition of multi-enzyme increased villus height and crypt depth, but these positive effects were not reflected in performance parameters in growing quails.

## Figures and Tables

**Table 1:** Composition of experimental diets and calculated nutrient contents.

Item	Sunflower Meal, %			
	0	10	15	20
Ingredients, g/kg				
Corn	537.0	495.0	473.0	452.0
Soybean meal	400.0	332.0	299.0	265.0
Sunflower meal	0.0	100.0	150.0	200.0
Sunflower oil	25.0	35.3	40.5	45.5
Limestone	10.6	10.5	10.4	10.2
Dicalcium phosphate	19.0	18.4	18.2	18.0
Salt	3.5	3.5	3.5	3.5
Premix ^1^	2.5	2.5	2.5	2.5
DL methionine	2.4	2.0	1.7	1.6
L Lysine	0.0	0.8	1.2	1.7
Calculated chemical composition				
Metabolizable energy, MJ/kg	12.13	12.14	12.15	12.15
Crude protein, g/kg	240.33	239.91	240.09	239.88
Ether extract, g/kg	50.73	49.63	64.11	68.41
Lysine, g/kg	13.15	13.05	13.03	13.09
Methionine, g/kg	5.25	5.27	5.20	5.29
Methionine + cysteine, g/kg	9.98	9.94	9.83	9.91
Calcium, g/kg	10.01	10.05	10.07	10.06
Non-phytate phosphorus, g/kg	5.01	5.00	5.02	5.03
Crude cellulose, g/kg	35.95	48.87	55.37	61.83

^1^ Vitamin–trace mineral mix supplied per kg diet: 80 mg manganese; 0.3 mg selenium; 5 mg copper; 40 mg iron; 0.15 mg iodine; 60 mg zinc; 12,000 IU retinol; 75 mg α-tocopherol acetate; 4000 IU cholecalciferol; 5 mg menadione; 3 mg thiamine; 6 mg riboflavin; 0.75 mg folic acid; 5 mg pyridoxine; 0.03 mg cyanocobalamin; 10 mg pantothenic acid; 40 mg nicotinic acid; 375 mg choline chloride; 0.075 mg D-biotin.

**Table 2:** Influence of sunflower meal (SFM) and multi-enzyme supplementation in diet on the performance parameters in growing quails *.

Treatment	BW, g/Quail			BWG, g/Quail/Period			FI, g/Quail/Period			FCR, g Feed/g Gain		
	Hatching	3 Week	6 Week	0–3 Week	3–6 Week	0–6 Week	0–3 Week	3–6 Week	0–6 Week	0–3 Week	3–6 Week	0–6 Week
**SFM, %**												
0	8.52	115.3 ^A^	206.1 ^A^	106.7 ^A^	90.9 ^A^	197.6 ^A^	307.1	387.6	694.8	2.88 ^B^	4.27 ^B^	3.52 ^C^
10	8.40	110.9 ^B^	203.3 ^A^	104.1 ^A^	92.4 ^A^	196.5 ^A^	305.4	388.4	693.8	2.93 ^B^	4.22 ^B^	3.53 ^C^
15	8.38	113.3 ^AB^	198.4 ^B^	104.9 ^A^	85.1 ^B^	190.0 ^B^	325.7	392.2	717.9	3.10 ^AB^	4.61 ^A^	3.78 ^B^
20	8.38	107.6 ^C^	189.0 ^C^	99.2 ^B^	81.4 ^B^	180.7 ^C^	329.0	395.7	724.7	3.33 ^A^	4.88 ^A^	4.01 ^A^
Pooled SEM ^1^	0.087	1.09	0.93	1.02	1.27	1.02	7.49	6.53	11.02	0.073	0.113	0.059
**Enzyme, g/kg**												
0.0	8.46	112.6	199.3	104.2	86.7	191.5	317.9	391.8	709.7	3.06	4.55	3.73
1.0	8.38	110.9	199.1	103.3	88.2	190.9	315.7	390.2	705.9	3.07	4.44	3.69
Pooled SEM ^1^	0.062	1.06	1.80	1.04	1.44	1.88	5.93	4.78	8.92	0.070	0.104	0.068
**SFM × Enzyme**												
0 × 0.0	8.38	114.2	206.5	105.8	92.3	198.1	304.3	379.0	683.3	2.88	4.11	3.45
0 × 1.0	8.67	116.4	205.8	107.7	89.4	197.1	310.0	396.3	706.3	2.88	4.43	3.58
10 × 0.0	8.54	112.8	204.4	104.2	91.6	195.9	306.1	387.3	693.5	2.93	4.24	3.54
10 × 1.0	8.25	109.0	202.3	101.4	93.2	197.2	304.6	389.5	694.1	2.93	4.19	3.52
15 × 0.0	8.46	113.8	197.4	105.3	83.6	189.0	330.2	399.5	729.7	3.13	4.79	3.86
15 × 1.0	8.29	112.8	199.4	104.5	86.6	191.1	321.2	385.0	706.2	3.08	4.44	3.69
20 × 0.0	8.46	109.9	189.1	101.4	79.2	180.6	330.9	401.4	732.3	3.28	5.08	4.06
20 × 1.0	8.29	105.4	189.0	97.1	83.6	180.7	327.0	390.1	717.1	3.38	4.69	3.97
Pooled SEM ^1^	1.000	1.40	1.30	1.41	1.54	1.23	11.06	8.38	14.05	0.107	0.137	0.075
**Probabilities**												
SFM	0.560	<0.001	<0.001	0.001	<0.001	<0.001	0.112	0.838	0.211	0.004	0.001	<0.001
SFM Linear	0.232	<0.001	<0.001	<0.001	<0.001	<0.001	0.029	0.382	0.052	<0.001	<0.001	<0.001
SFM Quadratic	0.462	0.558	0.003	0.191	0.053	0.001	0.763	0.847	0.758	0.307	0.152	0.105
Enzyme	0.329	0.127	0.824	0.441	0.245	0.557	0.792	0.823	0.764	0.918	0.314	0.592
SFM × Enzyme	0.097	0.166	0.545	0.294	0.246	0.765	0.973	0.380	0.575	0.912	0.111	0.428

BW: Body weight, BWG: Body Weight Gain, FI: Feed Intake, FCR: Feed Conversion Ratio, ^1^ Pooled Standard error of mean, * Data represent means based on four replicates per treatment, ten quails per replicate, ^A,B,C^ Within a column, means bearing different superscript are statistically different; *p* < 0.01.

**Table 3:** Influence of supplemental SFM levels and multi-enzyme addition in the diet on the meat yield and some relative organ weights in growing quails *.

Treatment	Carcass ^1^	Thigh + Drumstick ^2^	Breast ^2^	Liver ^1^	Heart ^1^	Pancreas ^1^	Gizzard ^1^	Proventriculus ^1^
**SFM, %**								
0	67.07	33.37 ^B^	55.88 ^A^	1.95 ^B^	0.946 ^A^	0.334	2.03 ^b^	0.394
10	68.08	33.33 ^B^	55.09 ^AB^	2.20 ^A^	0.963 ^A^	0.319	1.97 ^b^	0.374
15	66.91	33.61 ^B^	54.69 ^B^	1.78 ^B^	0.841 ^B^	0.307	1.99 ^b^	0.369
20	66.85	34.47 ^A^	53.61 ^C^	1.79 ^B^	0.993 ^A^	0.299	2.26 ^a^	0.376
Pooled SEM ^3^	0.863	0.228	0.357	0.084	0.0286	0.0199	0.084	0.0165
**Enzyme, g/kg**								
0.0	67.08	33.88	54.55	1.84 ^b^	0.926	0.302	2.03	0.368
1.0	67.37	33.50	55.08	2.01 ^a^	0.946	0.328	2.08	0.388
Pooled SEM ^3^	0.671	0.194	0.316	0.071	0.0239	0.0135	0.063	0.0118
**SFM × Enzyme**								
0 × 0.0	67.36	33.27	56.27 ^a^	1.75 ^BCD^	0.906 ^abc^	0.302	1.97 ^b^	0.343 ^b^
0 × 1.0	66.77	33.47	55.49 ^ab^	2.16 ^AB^	0.985 ^ab^	0.367	2.08 ^ab^	0.444 ^a^
10 × 0.0	66.78	33.66	55.18 ^ab^	2.20 ^A^	0.983 ^ab^	0.305	2.05 ^ab^	0.395 ^ab^
10 × 1.0	69.37	33.00	55.00 ^abc^	2.20 ^A^	0.943 ^abc^	0.333	1.89 ^b^	0.353 ^b^
15 × 0.0	67.12	33.92	53.84 ^bc^	1.90 ^ABCD^	0.891 ^bc^	0.315	2.09 ^ab^	0.363 ^b^
15 × 1.0	66.70	33.30	55.55 ^ab^	1.68 ^CD^	0.792 ^c^	0.299	1.88 ^b^	0.374 ^b^
20 × 0.0	67.07	34.67	52.93 ^c^	1.55 ^D^	0.922 ^abc^	0.285	2.02 ^ab^	0.371 ^b^
20 × 1.0	66.63	34.26	54.29 ^abc^	2.04 ^ABC^	1.064 ^a^	0.314	2.49 ^a^	0.382 ^ab^
Pooled SEM ^3^	0.955	0.298	0.414	0.082	0.0309	0.0252	0.093	0.0175
**Probabilities**								
SFM	0.881	0.007	0.001	0.001	0.002	0.639	0.030	0.684
SFM Linear	0.741	0.002	0.001	0.006	0.854	0.207	0.039	0.403
SFM Quadratic	0.666	0.067	0.648	0.080	0.017	0.853	0.033	0.385
Enzyme	0.817	0.120	0.114	0.015	0.440	0.201	0.480	0.202
SFM × Enzyme	0.756	0.544	0.033	0.002	0.014	0.568	0.012	0.024

^1^ % of body weight, ^2^ % of carcass weight, ^3^ Pooled Standard error of mean, * Data represent means based on four replicates per treatment, four quails per replicate, ^A,B,C,D^ Within a column, means bearing different superscript are statistically different; *p* < 0.01.^a,b,c^ Within a column, means bearing different superscript are statistically different; *p* < 0.05.

**Table 4:** Influence of SFM and multi-enzyme supplementation in diets on the histomorphology parameters of ileum in growing quails *.

Treatment	VW, µm	VH, µm	CD, µm	VH/CD	TMT, µm	VSA, mm ^2^
**SFM, %**						
0	43.57 ^C^	256 ^A^	40.54 ^B^	6.74 ^A^	24.38 ^A^	0.357 ^b^
10	53.84 ^A^	234 ^B^	37.82 ^C^	6.63 ^A^	22.27 ^B^	0.396 ^a^
15	47.12 ^B^	249 ^A^	48.49 ^A^	5.51 ^C^	21.72 ^B^	0.371 ^ab^
20	48.32 ^B^	231 ^B^	40.07 ^B^	6.11 ^B^	23.82 ^A^	0.360 ^b^
Pooled SEM ^3^	1.076	3.534	0.702	0.135	0.502	0.0106
**Enzyme, g/kg**						
0.0	49.08	237 ^B^	39.55 ^B^	6.36	24.14 ^A^	0.367
1.0	47.31	249 ^A^	42.92 ^A^	6.29	22.18 ^B^	0.376
Pooled SEM ^3^	0.788	2.557	0.498	0.100	0.349	0.0074
**SFM × Enzyme**						
0 × 0.0	45.14 ^BC^	255 ^A^	38.94 ^BCD^	7.06 ^AB^	23.37 ^A^	0.365 ^B^
0 × 1.0	42.44 ^B^	256 ^A^	40.54 ^BC^	6.51 ^BC^	25.10 ^A^	0.357 ^B^
10 × 0.0	50.24 ^B^	214 ^C^	38.28 ^CD^	5.92 ^CD^	24.24 ^A^	0.340 ^B^
10 × 1.0	58.06 ^A^	258 ^A^	37.82 ^D^	7.46 ^A^	19.95 ^B^	0.396 ^A^
15 × 0.0	50.56 ^B^	244 ^AB^	42.69 ^B^	5.98 ^CD^	23.79 ^A^	0.388 ^B^
15 × 1.0	43.51 ^BC^	254 ^A^	48.49 ^A^	5.02 ^E^	19.56 ^B^	0.354 ^B^
20 × 0.0	50.58 ^B^	240 ^AB^	39.04 ^BCD^	6.52 ^BCD^	25.21 ^A^	0.386 ^B^
20 × 1.0	46.07 ^BC^	227 ^BC^	41.11 ^BCD^	5.70 ^DE^	22.43 ^AB^	0.335 ^B^
Pooled SEM ^3^	1.507	4.848	0.959	0.187	0.683	0.0146
**Probabilities**						
SFM	<0.001	<0.001	<0.001	<0.001	<0.001	0.015
SFM Linear	0.179	<0.001	0.001	<0.001	0.441	0.591
SFM Quadratic	<0.001	0.842	<0.001	0.011	<0.001	0.011
Enzyme	0.102	0.001	<0.001	0.565	<0.001	0.373
SFM × Enzyme	<0.001	<0.001	<0.001	<0.001	<0.001	<0.001

VW: Villus Width, VH: Villus Height, CD: Crypt Depth, TMT: Tunica muscularis thickness, VSA: Villus Surface Area. ^1^ Pooled Standard error of mean, * Data represent means based on four replicates per treatment, one quail per replicate, ^A,B,C,D,E^ Within a column, means bearing different superscript are statistically different; *p* < 0.01. ^a, b^ Within a column, means bearing different superscript are statistically different; *p* < 0.05.

**Table 5:** Influence of SFM and multi-enzyme supplementation in the diet on the enzyme activities of the pancreas in growing quails *.

Treatment	Amylase, g/U	Lipase, g/U	Chymotrypsin, g/U
**SFM, %**			
0	84.29	1.134	0.319 ^A^
10	72.11	0.949	0.309 ^A^
15	89.26	1.128	0.188 ^B^
20	96.39	1.036	0.099 ^B^
Pooled SEM ^1^	8.961	0.1196	0.0411
**Enzyme, g/kg**			
0.0	95.50 ^a^	1.053	0.207
1.0	75.52 ^b^	1.071	0.250
Pooled SEM ^1^	6.302	0.0882	0.0380
**SFM × Enzyme**			
0 × 0.0	114.43 ^a^	1.462 ^A^	0.337
0 × 1.0	54.16 ^b^	0.806 ^B^	0.302
10 × 0.0	84.96 ^ab^	0.982 ^AB^	0.326
10 × 1.0	59.26 ^b^	0.916 ^AB^	0.293
15 × 0.0	82.24 ^ab^	0.889 ^AB^	0.070
15 × 1.0	96.27 ^ab^	1.367 ^AB^	0.305
20 × 0.0	100.38 ^ab^	0.878 ^AB^	0.095
20 × 1.0	92.40 ^ab^	1.194 ^AB^	0.103
Pooled SEM ^1^	9.591	0.1316^AB^	0.0418
**Probabilities**			
SFM	0.160	0.544	0.002
SFM Linear	0.122	0.804	<0.001
SFM Quadratic	0.207	0.653	0.041
Enzyme	0.013	0.864	0.298
SFM × Enzyme	0.013	0.003	0.090

^1^ Pooled Standard error of mean, * Data represent means based on four replicates per treatment, one quail per replicate, ^A,B^ Within a column, means bearing different superscript are statistically different; *p* < 0.01.^a,b^ Within a column, means bearing different superscript are statistically different; *p* < 0.05.
